# Extracts from a Discourse on the Disease Called Tic Douloureux, Read before the District Medical Society, at Boston, in April, 1812

**Published:** 1814-05

**Authors:** James Jackson


*For the Medical and Physical Journal.*

### *Extracts from a Discourse on the Disease called Tic Douloureux, read before the District Medical Society, at Boston, in April,* 1812; *by* James Jackson, M.D.

(*Concluded from p.* 187.)

### OF THE CURE.

DR. FOTHERGILL the elder, who was distinguished at once for humanity and professional skill, when he made his brethren acquainted with the character of this distressing disease, was at the same time enabled to direct them to a mode of relief, which in his hands had been almost uniformly successful. The medicine he employed was the hemlock (conium maculatum), an article which he was accustomed to administer more than most physicians of his own or any other age, and with the powers of which he was very well acquainted.

That eminent physician mentions three cases in which he employed this remedy with decided benefit; and says generally that after having once proved its efficacy, he always had recourse to it, and "for the most part with success."

Since his time, the character of hemlock, as an article of the materia medica, has very much depreciated. In regard to this disease, we can scarcely find on record a single case, in which this remedy has been thought to produce a cure, since Dr. Fothergill's publication on the subject. On the contrary, many declare expressly that the medicine has altogether failed to give relief. How are we to reconcile these contradictions?

As we must not too readily credit the account of any one man, who relates the virtues of a fashionable medicine; so, if such a man is of respectable character, we should not altogether distrust him, when the medicine has grown unfashionable. Dr. Fothergill could not be mistaken as to the facts, that his patients recovered of this obstinate disease, and that they recovered while using the hemlock. He might be in error when he attributed the recovery to the hemlock; but likewise he might have managed the remedy in some manner different from that of others.

It is remarkable respecting this remedy, more than respecting any other of which I know, that it produces different effects in different hands. I well remember that a very respectable physician told me, some years since, that he had seldom employed the hemlock with any advantage; but that one of his brethren in the neighbourhood had directed it in many cases with manifest benefit. These gentlemen are both both of the first rank among us, and particularly worthy of being so.

This statement will authorise me to inquire, what are the reasons for the different reports we have from those who have employed the hemlock?

The first reason is, the difference in the preparations employed. In a vast many cases, undoubtedly, the preparation which has been used has been entirely inert. The inspissated juice which has been commonly kept by our apothecaries, has been imported from England, and has been very badly prepared.*

But there are other reasons for the different effects of this medicine in the hands of different practitioners. Some, restrained by fear, not only begin with a very small dose, but increase it so very slowly, that in most instances the constitution becomes gradually accustomed to its effects; and, as happens to old smokers of tobacco, a very large dose may at length be borne without any very evident consequences. Others fail from want of confidence in the medicine: they prescribe it, but do not watch over the patient, and direct the use of it from day to day, so as to make it do all it is capable of doing.

These remarks lead us to consider what is the best mode of using the hemlock.

The object is, in most and perhaps in all cases, where this medicine is given, to produce a sensible effect on the system. The medicine may be beneficial without producing any such effect; but, if it is not beneficial, we cannot be said to give it a fair trial, until we have some proof that enough has been

* Several years since I requested Mr. Bartlett, druggist, of Boston, to have some of the inspissated juice prepared in his own shop. This was done, and he has every year since prepared a small quantity for those who were willing to pay the extra expence of it. This has been prepared with great accuracy, and I have had repeated proof of its superiority to that in common use. I will take the liberty to mention a case which will illustrate this. I prescribed the inspissated juice of hemlock for a patient affected with jaundice. In three days she had increased the dose to thirty grains without any effect. I then found that by sending to the wrong shop, she had procured some of the imported medicine, which, however, looked very well. I directed the medicine to be procured from Mr. Bartlett's shop, and the dose to be diminished. This last caution was a very necessary one. She took ten grains without any effect; but fifteen grains of the medicine prepared here were enough for her. After taking this dose she was unable to stand, or even set up for an hour or two, and from that time the yellowness of skin and all the other symptoms of her disease began to abate.

administered to produce an impression on the system. Now the quantity necessary for this purpose is very various. In some cases very small doses have been said to produce powerful effects. We are therefore obliged to begin with a small dose, and to increase this so far as the patient can bear it. It may be most prudent to begin with a single grain of the inspissated juice or extract, but then we may give five grains for the second or third dose; and afterwards we may add at least five grains to every dose, until we produce an effect on the system. But what are the effects on the system; and how soon will they appear? The most obvious effects of this medicine are displayed in its operation on the head and stomach. When given in a full dose, it occasions a slight nausea, a giddiness more or less severe, and often a loss of muscular power, so that the patient cannot stand up. It seldom does good, unless used in such a dose as to produce these effects in some degree, and indeed in as great a degree as can easily be borne.

These effects will begin to be evidenced in a very short time, if they are so at all. I will not venture to say precisely in what time; but I believe always in much less than two hours. Patients affected with neuralgia, who have taken this medicine during the paroxysms, have told me that when it produced any effect, this was manifested within fifteen or twenty minutes after swallowing it. I have therefore, in urgent cases, ventured to repeat the doses every half hour. I believe it must always be abundantly safe to repeat them once in two hours, if the patient and attendants are careful and watchful, and well-informed in regard to the effects.

My own experience of the efficacy of this remedy in neuralgia, conforms with that of Dr. Fothergill. It does not appear that he was obliged to give very large doses in any case; and it would seem that he did not repeat them oftener than twice in twenty-four hours. But he did not consider himself as having given the medicine a trial, until he had administered enough to produce evident effects on the system. For the want of success of others since his time I cannot offer any explanation, except that contained in the foregoing remarks.

In certain cases the sensibility of the diseased nerves had been destroyed by dividing the trunk of the particular nerve, from which the diseased branches proceed.

It is remarkable that this practice was conceived and adopted in both France and Great Britain, without being derived from one country to the other. A French surgeon, by the name of Mareschal, is said to have been the first who adopted this mode of treatment on the continent of Europe.

In England it was first proposed and adopted by Dr. Haighton, after he had discovered the true seat of the disease.

In the case* in which Dr. H. adopted this practice, the suborbitar nerve was diseased; and he divided this by one incision extended to the bone, and made at the distance of one quarter of an inch from the suborbitar foramen. Dr. H. thought the same mode of treatment might be pursued where the frontal branch of the first branch of the fifth pair was the seat of the disease; but he did not think this treatment applicable where the disease affected the third branch of the fifth pair, or the portio dura of the seventh pair of nerves. It appears, however, by a statement in Darwin's Zoonomia, that under the direction of Mr. Cruikshank almost every nerve of the face was divided in one instance, and with eventual success.

This mode of treatment is certainly to be advised in cases where the disease resists internal remedies. It is not infallible, for in some instances the nerve uniting, the pain has returned in a short time; in others, after a number of years.

I ought, perhaps, to add, that in France they have employed not only the knife, but also caustic, to destroy the nerve. This operation must be more painful than the incision, and at the same time less certain and less safe. For instance, the suborbitar nerve lies directly upon the bone: now it must be almost impossible to destroy this nerve by caustic without injuring the bone.

To both modes of division it has been objected that the parts which are furnished by the divided nerves will be paralized. In severe cases this cannot be a sufficient objection to the practice, since the same parts are worse than paralised by the disease. Besides, it is known that the nerves will re-unite, and their functions be restored in a short time.

It has been found that various stimulants have been usefully employed in the treatment of this disease. I must be brief in noticing them.

Electrical sparks and other local stimuli have afforded some mitigation in this most distressing complaint; and in one case electricity was thought to effect a cure.

Aqua Ammoniæ† was successful in the hands of Dr. S. Fothergill. I have tried the same, and once with success.

---

* In this case the disease returned at the end of some years as stated by S. Fothergill, but with less violence. In other cases, however, it has not returned; in some it has. Yet, when it does, the operation may easily be repeated in case of the suborbitar nerve.

† Aq. Ammon. caust. vel pur.

Preparations

Preparations of zinc, silver, quicksilver, and arsenic, have been found useful in some instances. Two cures were effected by mercurial salivation in France. The disease was supposed to have had a syphilitic organ; but the evidence in support of this opinion is not conclusive. In other cases the use of mercurials has been thought to aggravate the disease. Arsenic is perhaps more worthy of further trial than any of the metallic remedies;—but in the present state of our knowledge it appears to me that the use of the narcotics deserves a fair experiment before any of those remedies.

I shall now, gentlemen, only beg your patience for a short time longer, while I read to you the cases of this disease which have fallen under my observation, and I will not detain you even to apologise for the undue length to which this discourse has been protracted.

*Case of Mrs. O.*—This patient came under my care about four years since. She was past sixty years of age. She had previously been subject to severe colic, fits of which were produced whenever she ate any flatulent food, particularly beans. This disease was, perhaps, connected with some hepatic affection, or with gall-stones, for one of the fits of colic was followed by jaundice.

When I saw her, she had been suffering with the pain in her face for about two years. The infra-orbitar nerve was distinctly the seat of the disease. The pain was often induced by a very slight touch, though she could bear hard pressure on the part. The disease was of the most unequivocal character.

I administered the aq. ammoniæ, the use of which had then been recently recommended by Dr. S. Fothergill. The dose was at first one tea-spoonful, but this was gradually increased to three tea-spoonfuls, and was administered three times a-day. This disease shortly after gave way, and the patient had a respite of some months; a respite longer and more perfect than she had enjoyed before, since the disease commenced. Yet at first I thought this might be accidental. At length the pain recurred with less severity, and yielded at once to the same remedy. I am not sure that it did not recur once more, slightly. The patient promised to let me know if the disease ever appeared again, and as she was very grateful for the relief she received, it is probable she would not forget this promise. It is now three years since I have seen her, and therefore I presume she continues well. I have lately inquired for her, and know that she is living, but I could not find her.

*Case of Mrs. W.*—This lady applied to me in the autumn of 1808, when she was about twenty-five years old. She had for some years been afflicted with pain on the right side of her face, which she had referred to her teeth, and all those in the upper jaw on that side, except the incisors, had been removed, with a hope of obtaining relief. The character of these pains, previous to the autumn of 1808, is uncertain. The pain was called *tooth-ache* at the time; but it must at least have been very severe to induce a young and handsome woman to give up so many teeth.

When Mrs. W. first applied to me, she had been suffering extreme pain for a month. The pain came on in the night much more than in the day, though not at regular hours. This was unlike the neuralgia. After I visited her, it occurred very often in the day, and gradually became less frequent in the night. The most constant seat of the pain was in the course of the sub-orbitar nerve; passing from the alveolar processes, the lips, cheek, and side of the nose, to the eye; that is, from the branches to the trunk of the nerve. When severe, it often extended itself, passing into the lower jaw, to the ear, over the forehead, and to the side of the head. The severe paroxysms were attended with a swelling of the gums of the upper jaw, which from the feelings of the patient seemed to be very considerable, but which on examination appeared to be very slight. The pain was sudden in its access, violent, darting, shooting, and consisted in strokes following each other in quick succession. Its continuance was various, but the dread of it was horrible; and the patient seemed unable to convey an idea of her agony. During the paroxysms the patient was quite unable to take food, and was very unwilling to speak. She tried to keep the cheek and jaw on the right side quiet, while she was speaking; yet the paroxysms were not often produced by eating or speaking.

I first gave a fair trial to the aqua ammoniæ. The dose was gradually increased to three tea-spoonfuls, and was administered three times a-day. Although the occasional remissions which always occur in this disease encouraged us to go on, yet eventually it appeared that this remedy was not adequate to the cure of the disease.

I then directed the conium maculatum, of which the inspissated juice (extract) was given twice a-day. The dose, which at first was small, was gradually increased, and it was repeated more frequently. Now and then it seemed to give relief, and the head would be a little affected. But the system seemed to become accustomed to the medicine very rapidly.

I conceived

I conceived that if the system could be made to feel the effects of this medicine suddenly and powerfully, there would be a chance of producing a real cure; a better chance than from giving a much larger quantity during a long space of time. Accordingly, after having exhibited the medicine for about a fortnight, and increased the dose to six and eight pills of five grains each; and having ascertained that whenever any sensible effect was produced, it took place within fifteen or twenty minutes after the dose was swallowed, I resolved on a bold trial of this medicine. I directed eight pills to be taken as soon as the pain should come on, and that the same should be repeated, at the end of twenty-five or thirty minutes, if the pain should continue, and the medicine should not affect the head or stomach. At this time the paroxysms of pain had become inexpressibly severe and very frequent. My directions were carefully followed. The intervals of pain were such as sometimes to prolong the intervals between the doses; but in the course of six hours, sixty* pills were taken, making three hundred grains. After the last dose, the patient was quite overcome by the medicine. She became dizzy and faint, and was unable even to sit up. She laid upon a sofa for some time, in a state of intoxication, but without suffering any very unpleasant effects afterwards. The respite from pain and from susceptibility of pain, was more perfect and longer than at any time for several weeks before. But the disease was not conquered: it returned the next day with considerable severity. I had directed the medicine to be repeated as soon as the pain should return, which was done, and in the course of the day forty-eight pills were swallowed. These gave entire relief, and affected the head, but not so powerfully as on the day before. The disease seemed now to be vanquished. This was in November 1808, and for two months the pain was not felt, except in very transient twinges, which were never repeated for any length of time. About the middle of January following, this lady walked out on an extremely cold day, and the disease returned before night with very considerable force. The hemlock was employed again, but in smaller doses at first. In three days the disease gave way, without having required very large doses of the medicine. Since that time it has never recurred; that is, during more than three years.

*Case of Miss S.*—I saw this lady in June 1809, when I learnt the following history. In the early part of the pre-

* The patient could not every time get down the whole of the eight large pills, so that some doses were seven and some eight; whence the whole came to be sixty.

ceding winter, she began to be affected with a pain in her face, without any preceding ill health. At this time she was not quite twenty years of age. The pain continued to trouble her excessively through the winter and spring. The principal seat of pain was the lower jaw, going back to the ear; but it often spread over the cheek, and sometimes to the temple. It was not in the teeth, for there was not any diseased tooth near the parts affected, and the pain was referred to the external parts, not to the bone of the jaw. This pain was described as excruciating in the highest degree, and as coming on suddenly in shoots and strokes.

I directed the tincture of hemlock;* thirty drops for the first dose; but ordered that the dose should be frequently repeated, and presently increased if relief were not obtained.

The patient took two doses, thirty drops each, the evening after I had ordered it, with great benefit. After this she was able to command the pain, but was obliged for this purpose to increase the dose very much. In a short time the pain left her. I did not see her for many months after my first visit in June; but learnt that she had a return of pain in the October following, which was relieved by hemlock.

Two years afterwards the disease recurred, viz. in August 1811, when she had been married, and was nursing a child then about four months old. The return of the disease was perhaps owing to debility, occasioned by the warm weather and nursing, which her system did not appear to support with ease. She again took the tincture of hemlock, but unfortunately was obliged to go out of town, so that I could not attend to the administration of the medicine. The dose was gradually increased to three hundred drops. It never gave relief except when it affected her head, and always when it did, as she afterwards told me. The disease yielded in two or three weeks after she commenced the use of the hemlock, and since that time it has not returned.

*Case of Mrs. C.*—This is a lady now in her 74th year, who has enjoyed unusual health through her life, and even now retains uncommon vigour, notwithstanding she has undergone

* This was prepared in the same proportions as the tinct. digital. p. I have known it doubted whether the medicinal virtues of the hemlock are given out to proof-spirit. On this head I have obtained satisfactory evidence. In the above case, it will be seen that the head was affected, as when other preparations of hemlock are given. I have twice given this preparation in cases of icterus, as proposed by Dr. Fisher, and with unequivocal success. In one of these cases the head was made dizzy, and relief obtained by twenty-five drops; but in general much larger doses are required.

dergone extreme pain in her face for a long time. I was called to her on the 21st of February last. I found her under the utmost terror of the paroxysms of neuralgia of the face, so that she was unwilling to speak to me except in monosyllables, and the shortest sentences.

Her history was briefly this. In November 1809, she first experienced a pain in the upper jaw of the right side, not in a tooth nor socket, for she had lost all her teeth many years ago. This pain occurred when she was eating, but not from biting any thing unusually hard. She felt as if something gave way. Soon afterwards this pain occurred, and became more and more frequent and more severe. For many months (until the ensuing summer) she suffered excruciating pain, which was not confined to the jaw, but spread over the cheek, and at length to the whole side of the head, to the lower jaw, and to the palate. This pain was not constant; it was sudden in its occurrence and often so in its departure, of uncertain duration and frequency. It was brought on by the slightest touch in many instances, such as adjusting her cap, and even by the warmth of a hand approaching her. It was very often brought on by speaking and by eating, so that she kept herself as silent as possible, and has lived on a very small quantity of food, not thinking of gratifying her appetite. At one time she is said to have passed a fortnight almost without sustenance.

In this case the pain occurred equally in night and day. It was attended by a swelling of the gums in the jaw affected, and at times by a redness of the cheek. It was also accompanied by a hacking cough, as if the disease extended to the borders of the larynx.

Since the spring of 1811, she has been less frequently and less severely affected; but for the two or three weeks before I saw her the pain had been increasing in severity, and threatened her with sufferings as excruciating as heretofore.

This lady has had the advice of two of the most respectable, and the two oldest physicians now living in this place. Various remedies were ordered by them, but without benefit, as the patient reports.* I ordered the inspissated juice of the hemlock to be taken night and morning, beginning with one pill of five grains, and adding one pill to each dose. I intended, however, to administer this medicine more freely after seeing how the patient bore it. In three days the patient began to experience some relief, and in eight days she

* I have learnt since that the conium mac. was ordered, but from some circumstances never taken, at least only three pills, which the patient fancied to hurt her.

declared declared that she was almost free from pain, though the parts continued very sensible. From that time she has not had any pain of any consequence, and considers herself almost as one restored to life, as she had, before using the hemlock, quite despaired of ever obtaining relief from sufferings too severe for utterance.† —*New-England Journ. of Med. and Surg. April* 1813.

† This lady had one slight attack in May or June last, and was immediately relieved as before. The other patients have not had any return of the disease. March, 1813.

